# Diverse Hematological Malignancies Including Hodgkin-Like Lymphomas Develop in Chimeric MHC Class II Transgenic Mice

**DOI:** 10.1371/journal.pone.0008539

**Published:** 2009-12-31

**Authors:** Silke H. Raffegerst, Gabriele Hoelzlwimmer, Sandra Kunder, Josef Mysliwietz, Leticia Quintanilla-Martinez, Dolores J. Schendel

**Affiliations:** 1 Institute of Molecular Immunology, Helmholtz Zentrum München, German Research Center for Environmental Health, Munich, Germany; 2 Clinical Cooperation Group Immune Monitoring, Helmholtz Zentrum München, German Research Center for Environmental Health, Munich, Germany; 3 Institute of Pathology, Helmholtz Zentrum München, German Research Center for Environmental Health, Neuherberg, Germany; Health Canada, Canada

## Abstract

A chimeric HLA-DR4-H2-E (DR4) homozygous transgenic mouse line spontaneously develops diverse hematological malignancies with high frequency (70%). The majority of malignancies were distributed equally between T and B cell neoplasms and included lymphoblastic T cell lymphoma (LTCL), lymphoblastic B cell lymphoma (LBCL), diffuse large B cell lymphoma (DLBCL), the histiocyte/T cell rich variant of DLBCL (DLBCL-HA/T cell rich DLBCL), splenic marginal zone lymphoma (SMZL), follicular B cell lymphoma (FBL) and plasmacytoma (PCT). Most of these neoplasms were highly similar to human diseases. Also, some non-lymphoid malignancies such as acute myeloid leukemia (AML) and histiocytic sarcoma were found. Interestingly, composite lymphomas, including Hodgkin-like lymphomas, were also detected that had CD30^+^ Hodgkin/Reed-Sternberg (H/RS)-like cells, representing a tumor type not previously described in mice. Analysis of microdissected H/RS-like cells revealed their origin as germinal center B cells bearing somatic hypermutations and, in some instances, crippled mutations, as described for human Hodgkin lymphoma (HL). Transgene integration in an oncogene was excluded as an exclusive driving force of tumorigenesis and age-related lymphoma development suggests a multi-step process. Thus, this DR4 line is a useful model to investigate common molecular mechanisms that may contribute to important neoplastic diseases in man.

## Introduction

The similarities between genomes and genetic pathways underlying tumor development in mice and humans make mouse models ideal for the study of cancer pathogenesis. Numerous inbred, virus-induced or genetically-engineered mouse models of human malignancy have been developed in the past to gain insight into mechanisms of tumorigenesis.

Several mouse models exist for hematopoietic malignancies which correspond to distinct tumor entities, including B or T cell lymphomas or myeloid leukemias [1], [2]. A number of these models rely on tumor transplantation, which poorly reflects the process that occurs in human malignancy, particularly with respect to multi-step events. This can be partially rectified with the use of spontaneous tumor models that are created by introducing known genes, such as chromosomal translocations which occur in human tumors. As examples, B cell malignancies were induced by translocation of the myc-locus [3], knock-in of the proto-oncogene Bcl-6 [4] or knock-out of the p53 tumor suppressor gene [5]. Spontaneous T cell malignancies were generated by expression of mutated Notch1 [6], in Emu-myc transgenic mice [7] or by induction of Moloney mouse leukemia virus [8]. Furthermore, leukemic malignancies were forced through integration of fusion-genes that mimic chromosomal alterations, such as myc-IgH [9], CALM/AF10 [10] or through introduction of murine leukemia viruses (MuLV) [11]. These various models share the characteristic that principally one distinct lineage-specific tumor-type is detected in each mouse strain.

We discovered that an established MHC class II transgenic (tg) mouse line, developed by Ito and coworkers as a model for autoimmunity [12] developed spontaneous tumors starting around eight months of age. Extensive cellular and molecular characterization revealed that widely diverse hematopoietic neoplasms occur in these mice, which arise from different lineages and represent different stages of lymphocyte development. Surprisingly, many of these neoplasms had phenotypes that are characteristic of human hematological diseases. Therefore, this mouse line provides a unique tumor model that broadly extends the possibilities to study potential multistep-processes involved in tumorigenesis in vivo, with high relevance to human hematological malignancy.

## Results

### Various Hematological Malignancies Develop in DR4-H2E and F1 Mice

The previously published DR4-H2E transgenic mouse line expresses a chimeric human-mouse MHC class II gene (HLA-DR4/H2E^d^). DR4-H2E mice express MHC class II chimeric molecules, composed of the human DR4α and DR4β peptide binding-domains, coupled to mouse MHC class II (H2Eα**^d^** and H2Eβ**^d^**) constant domains, respectively. One DR4-H2E transgenic founder line was backcrossed to MHC class II-deficient mice (C2d–C57BL/6 background) to eliminate expression of endogenous mouse class II molecules and DR4-H2E homozygous mice (hereafter DR4 mice) were established [12]. All offspring of DR4 parental mice expressed chimeric class II molecules on splenic B cells but not T cells, as depicted using a monoclonal antibody specific for human DRB1*0401 molecules (data not shown). These results confirmed homozygosity of the DR4-H2E transgene in the parental line. Cellular interactions were preserved with CD4 coreceptors on murine T cells by retaining the alpha-2 and beta-2 domains of mouse MHC class II in the chimeric protein.

**Figure 1:**
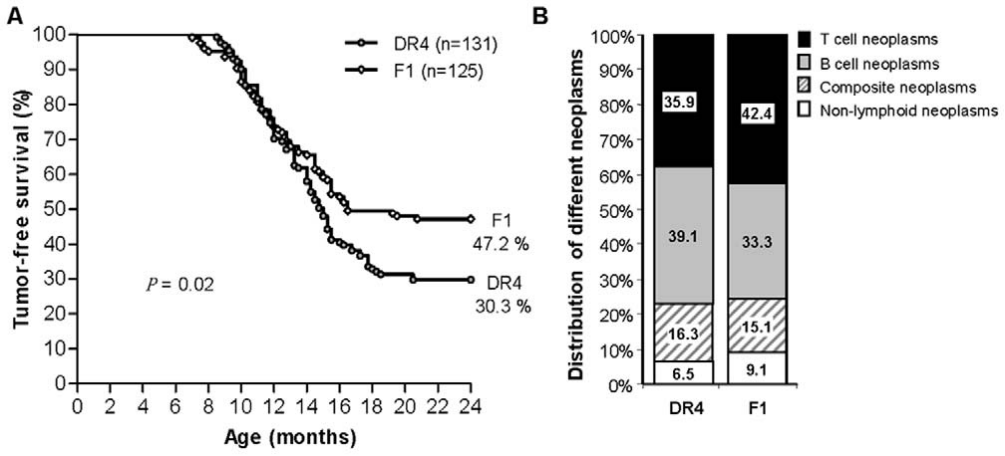
Survival plot and comparison of neoplasm distribution between DR4 and F1 mice. (A) Tumor-free survival of DR4 and F1 mice within 24 months shown by the frequency of diseased mice in DR4 and F1-strains in relation to their age. Each symbol represents one individual mouse. Disease appeared at 8.5 months of age in DR4 mice whereas in the heterozygous F1 generation tumors could be observed at 7 months. Around 70% of DR4 mice and 53% of F1 mice developed lymphomas, 30% and 47% survived, respectively. Results were subjected to statistical analysis using GraphPad Prism v5.0 software (GraphPad Software, San Diego California USA, www.graphpad.com). Survival curves were analyzed using the Kaplan-Meier method and were compared with the log-rank test and by median survival. Kaplan-Meier survival curve shows significantly decreased median survival of DR4 compared with F1 mice. Median survival of DR4 was 16.5 months versus 15 months for F1-mice (log-rank test, p = 0.0159). Data represent DR4, n = 131; F1, n = 125. (B) Distribution of different neoplasms in DR4 mice compared with F1 mice. Both mouse strains developed lymphoid tumors (T and B cell neoplasms) with a similar frequency of around 75%.

Our new finding is that DR4 mice spontaneously develop hematopoietic malignancies at high frequency (69.7%). To explore the role of DR4 homozygosity versus murine H2 class II deficiency on tumor occurrence, DR4 animals were crossed with wildtype C57BL/6 mice, yielding DR4 heterozygous F1 mice which carry one normal H2^b^ MHC haplotype and thereby are no longer H2 class II deficient. Tumor development in F1 mice would imply a dominant oncogenic effect associated with DR4-H2E transgene heterozygosity and only a minor contribution of H2 class II deficiency to tumorigenesis. Indeed, DR4 heterozygous F1 mice also developed different types of lymphoma with high frequency, as described in more detail below. Tumor appearance was assessed in 132 DR4 and 125 F1 mice, yielding frequencies of 69.7% (92 of 132) and 52.8% (66 of 125), respectively (Figure 1A).

Tumors were classified histologically according to the Bethesda Proposal for lymphoid and non-lymphoid hematopoietic neoplasms in mice [1], [2]. Expression of lineage-specific markers was assessed by immunohistochemistry to support histopathology and was confirmed in many cases by multi-parameter flow cytometry of thymic or splenic single cell suspensions. The hematologic neoplasms arising in DR4 and F1 mice were similar (Figure 1B). Most malignancies were of lymphoid origin with 35.9% (33 of 92) T cell tumors and 39.1% (36 of 92) B cell tumors in DR4 mice (Table 1). Several cases of AML (4.3%) and two cases of histiocytic sarcoma were also observed. Furthermore, 16.3% of animals had composite tumors with mixtures of B cell and T cell tumors or B cell tumors with AML (Figure S1). Some composite tumors contained mononucleated Hodgkin-like or multinucleated RS-like cells (see below). These lymphomas accounted for 10.9% of malignancies and were generally a mixture of T cell/histiocytic rich variant of DLBCL and LTCL. A similar occurrence of HL in composite lymphomas appearing with LBCL [13], with the T cell rich variant of DLBCL [14] or with MZL [15] has been described previously in man.

**Table 1:** Distribution of neoplasms in transgenic DR4-mice.

Type of neoplasm	Number	Frequency (%)
**Lymphoblastic T cell lymphoma**	**33**	**35.9**
Precursor lymphoblastic T cell lymphoma (CD4^+^CD8^+^ LTCL)	10	10.9
CD4+ T cell lymphoma (CD4^+^ LTCL)	15	16.3
CD8+ T cell lymphoma (CD8^+^ LTCL)	2	2.2
LTCL with undefined phenotype (no samples for FACS)	6	6.5
**B cell neoplasms**	**36**	**39.1**
Lymphoblastic B cell lymphoma (LBCL)	4	4.3
Diffuse large B cell lymphoma (DLBCL) (incl. 1x case with H/RS-like cells)	6	6.5
Diffuse large B cell lymphoma, rich in histiocytes (DLBCL-HA) = T cell rich diffuse large B cell lymphoma (incl. 3 cases with H/RS-like cells)	6	6.5
Splenic marginal zone lymphoma (SMZL)	12	13.0
Follicular B cell lymphoma (FBL)	5	5.4
Plasmacytoma (PCT)	2	2.2
B natural killer cell lymphoma (BNKL)	1	1.1
**Composite neoplasms**	**15**	**16.3**
LTCL+DLBCL-HA (incl. 5 cases with H/RS-like cells)	7	7.6
SMZL+DLBCL-HA (incl. 1 case with H/RS-like cells)	1	1.1
DLBCL+LTCL (incl. 1 case of histiocytic sarcoma)	2	2.2
LBCL+LTCL	1	1.1
DLBCL+acute myeloid leukemia (AML)	1	1.1
LBCL+AML	1	1.1
SMZL+AML	1	1.1
SMZL+histiocytic sarcoma	1	1.1
**Non-lymphoid neoplasms**	**6**	**6.5**
AML	4	4.3
Histiocytic sarcoma	2	2.2
**Other malignancies**	**2**	**2.2**
Epidermal tumor	2	2.2
**Number of diseased mice in study**	**92**	**100**
Disease-free mice	40	
**Total number of mice in study**	**Σ132**	

### Characterization of T Cell Neoplasms

**Figure 2:**
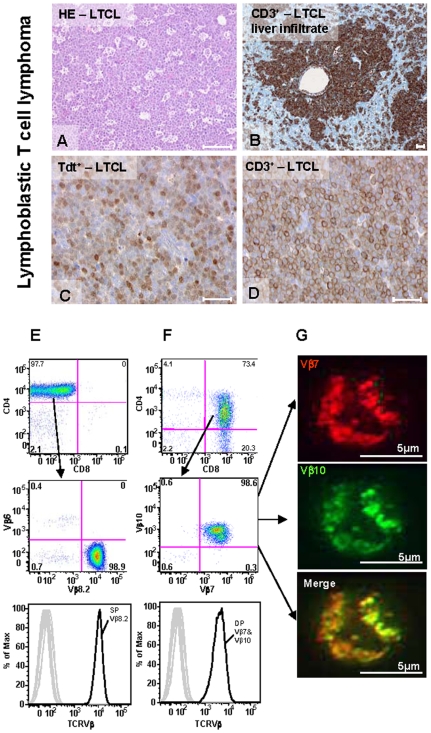
Lymphoblastic T cell lymphoma (LTCL). (A–D). Histology and immunohistochemistry of LTCL (all bars equal 50 µm). (A) LTCL consists of uniform cells with scant cytoplasm showing high mitotic and apoptotic rates and the typical starry sky pattern (hematoxylin & eosin, H&E). (B) CD3+ infiltrates in the liver. (C). Tumor cells are positively stained for Tdt (terminal deoxynucleotidyl transferase) and (D) CD3. (E–F) Flow cytometry analysis of T cell receptor Vβ-repertoire on tumor-bearing spleen samples. (E) Monoclonal single positive (SP) CD4+ LTCL. Dot plots - top: gated on CD3+B220− cells: CD4 versus CD8, middle: gated on SP CD4+ T cells showing the Vβ8.2+ tumor cell population and an irrelevant Vβ-chain (Vβ6); bottom: gated on DP CD4/Vβ8.2 as shown in histogram all other Vβ-families were negative. (F) Monoclonal precursor LTCL gated on CD3+ B220− cells: top: CD4 versus CD8, middle: monoclonal DP CD4+CD8+ tumor cells expressing two Vβ-chains (Vβ7&Vβ10), bottom: gated on DP Vβ7/Vβ10 tumor cells, as shown in histogram all other Vβ-families were not expressed. (G) Lymphocytes were stained as for FACS-analyses; top: Vβ7-PE in red, middle: Vβ10-FITC in green, bottom: merged image showing co-localization of Vβ7-PE and Vβ10-FITC as dual T cell receptors on splenic tumor cells with deconvolution microscopy. All bars equal 5 µm.

All murine T cell neoplasms were diagnosed as lymphoblastic T cell lymphomas (LTCL). These had a typical “starry sky” pattern (Figure 2A) with massive infiltration of lymph nodes, spleen, lung, kidneys and liver (Figure 2B). These neoplasms represented a monotonous population of medium-sized cells, with scant cytoplasm and blastic chromatin with one to two small nucleoli, intermingled with abundant histiocytes with tingible bodies. Immunohistochemical analysis revealed that neoplastic cells were terminal deoxynucleotidyl transferase (Tdt)- and CD3-positive (Figure 2C–D) but negative for the B cell marker, B220. LTCL were either single-positive (SP) CD4^+^ or CD8^+^ T cells (Figure 2E) or double-positive (DP) CD4^+^CD8^+^ T cells (Figure 2F), as shown by flow cytometry. Single-positive LTCL represent a common type of lymphoma in mice that is not found in humans [16], whereas DP LTCL have recognized human counterparts.

Molecular analysis of T cell receptor (TCR) usage indicated a monoclonal origin of all T cell tumors that were examined in DR4 and F1 mice (Table S1 and Table S2). To assess clonality, we used an RT-PCR-based TCR Vβ-repertoire analysis detecting 21 Vβ-families [17]. All detected amplicons were sequenced and evaluated for standard characteristics of functionally rearranged Vβ–chains: in-frame rearrangements, lack of stop-codons, conserved cysteine at position 104 and the conserved Phe-Gly-X-Gly-motive (IMGT-Database). Where possible, molecular characterization of LTCL was supported by TCR surface phenotyping of neoplastic cells using corresponding Vβ-specific monoclonal antibodies. The frequency of tumor cells in starting materials of LTCL varied from 36–99%, perhaps reflecting the status of tumor progression in the examined animals (data not shown). Despite cellular heterogeneity, the TCR Vβ-repertoires of all tumors were restricted to a few prominent amplicons. Furthermore, in all cases but one (i.e. Mouse ID-10), only a single amplicon sequence displayed the characteristics of a functional Vβ-chain. In several tumors, a second amplicon revealed a “non-functional” rearranged TCR sequence. The presence of some contaminating normal T cells was evident in the background of electropherograms, but these sequences were below a signal threshold that allowed individual sequences to be defined, suggesting they were composed of mixtures of different sequences (data not shown). As examples, Figure 2E shows a monoclonal SP CD4^+^ tumor expressing a Vβ8.2-receptor representing 98% of splenic CD4 T cells. The surface phenotype confirmed the TCR sequence designation of this tumor (Mouse ID-8, Table S1). In the second case, two Vβ-receptor sequences were found by RT-PCR (Mouse ID-10, Table S1) and surface phenotyping of DP CD4^+^CD8^+^ tumor cells showed staining with corresponding Vβ-specific monoclonal antibodies (Figure 2F). Confocal microscopy corroborated that both Vβ-chains were colocalized on individual cells (Figure 2G). While human T cells naturally expressing two Vβ-chains have been reported previously [18], this has not been seen with normal mouse T cells but has been observed for T cells from TCR transgenic animals that co-express endogenous TCR [19]. Based on combined molecular and cellular analyses, the results indicated a monoclonal origin of all T cell tumors that were examined in DR4 and F1 mice (Table S1 and Table S2).

### Characterization of B Cell Neoplasms

**Figure 3:**
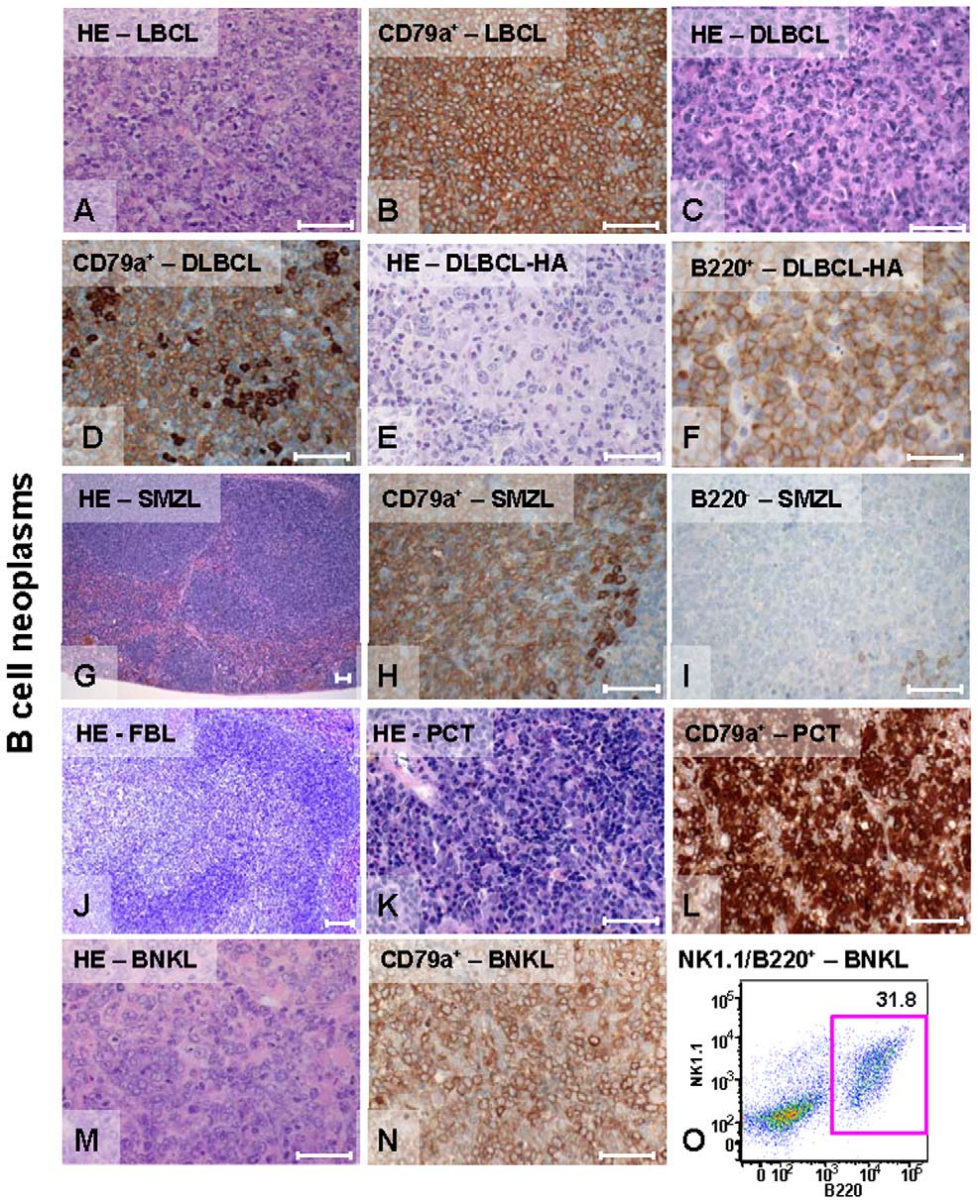
B cell neoplasms. (A–K) Histology (H&E) and immunohistochemistry (all bars equal 50 µm). (A) Lymphoblastic B cell lymphoma (LBCL) shows blastic nuclei with prominent nucleoli and numerous mitotic figures. (B) Staining for CD79a+. (C) Diffuse large B cell lymphoma (DLBCL) consists of immunoblastic and centroblastic cells and is (D) CD79a+. (E) The histiocyte-associated variant (DLBCL-HA) shows participation of many histiocytes and granulocytes. (F) B220+ DLBCL-HA. (G–I) The splenic marginal zone lymphoma (SMZL) is usually (H) CD79a+ but (I) B220−. (J) H&E of follicular B cell lymphoma. (K–L) Well-differentiated plasmacytomas display (L) CD79+ plasma cells. (M–O) The cellular phenotype of the B natural killer cell lymphoma (BNKL) is confirmed by (N) positively stained CD79a B cells and (O) DP B220/NK1.1 cells in FACS.

The B cell neoplasms were morphologically very heterogeneous but all were positive for CD79a (Igα) and/or B220. B cell lymphoblastic lymphomas (LBCL) (Figure 3A+B) were characterized by medium-sized cells with scant cytoplasm, blastic chromatic and one prominent nucleolus. These tumors showed massive infiltration of different organs, starry sky patterns and numerous mitotic figures. Tumor cells were positive for CD79a (Figure 3B), B220 and Tdt. Diffuse large B-cell lymphomas (DLBCL) (Figure 3C+D) had medium to large centroblastic and immunoblastic cells with pale cytoplasm, round vesicular nuclei and prominent nucleoli. Many DLBCL showed conspicuous infiltration with reactive CD3-positive T cells. Half of the DLBCL belonged to the morphological histiocyte-associated variant DLBCL-HA (Figure 3E+F), which corresponds to T cell/histiocytic rich variant of DLBCL in man [20]. Splenic marginal zone lymphomas (SMZL) (Figure 3G+I) were composed of monomorphic, pale tumor cells with regular nuclei and abundant cytoplasm. Early cases were restricted to the marginal zone and advanced cases showed invasion into the red pulp of the spleen or rarely into other organs. Their low proliferative activity was reflected by a low number of mitotic figures (data not shown). SMZL were always CD79a positive (Figure 3H) and often negative for B220 (Figure 3I). The similarity of SMZL to its human counterpart is very high. The murine follicular B cell lymphoma (FBL) was characterized by a mixture of centrocytes and centroblast cells (Figure 3J). These tumors were usually B220 positive and negative for Bcl2 (data not shown). Although morphologically similar to human lymphomas, the pathogenesis of murine FBL seems to be different [21]. Well-differentiated plasmacytomas (PCT) (Figure 3K+L) consisted of plasma cells in different stages of maturation, which were positive for CD138 and CD79a. One case of B natural killer cell lymphoma (BNKL) was also found (Figure 3M–O). It consisted of large pale cytoplasm-rich cells, often with indented nuclei and prominent nucleoli. Tumor cells appeared as mature large B cells with surface expression of CD79a (Figure 3N) and NK1.1 in FACS (Figure 3O). This tumor lacks any known human counterpart.

FACS analysis confirmed a mature (B220^+^IgM^+^IgD^+^) phenotype of most B cell tumors, except LBCL. A few lymphomas had phenotypes of small pre-B cells (Mouse-ID 96,124; B220^+^Ig^−^CD117^−^), pro B-cells (Mouse-ID 109; B220^+^Ig^−^CD117^+^) or immature B cells (Mouse-ID 48,111,129; B220^+^IgM^+^IgD^−^) (Table S3 and/or Table S7). To determine clonality, RT-PCR analysis was made with immunoglobulin heavy chain (IgH)-specific primers for variable (V) and joining (J)-regions. Clonality was identified by sequence homogeneity of the amplified IgH fragments. Since whole spleen samples were used, a low background of contaminating sequences from normal B cells was observed but these mixed signals were below the threshold allowing individual sequence determination (data not shown). Sequences were considered to be monoclonal and functional if they were in-frame, had no stop-codons, a conserved cysteine at position 104 (IMGT-Database) and a Trp-Gly-X-Gly-motive similar to TCR analysis. Results indicated monoclonality in 80.6% of DR4 tumors and 78.6% of F1 tumors. In the remaining samples, the VDJ-junctions were not discernable as they consisted of multiple sequences, indicating that these tumors were likely to be oligoclonal. Further analysis of sequence data in comparison to reported germline sequences [22] was performed to search for mutations (silent or replacement mutations) in the framework regions FR1-FR3 and the complementarity determining regions CDR1–CDR3. The presence of mutations in FR1–FR3 and CDR1–CDR2, in addition to mutations in the CDR3-regions of VDJ-junctions, would indicate that tumor cells originated from mature, activated B cells of germinal centers with ongoing somatic hypermutations (SHM). Thirty percent of the examined DR4 B cell tumors (9 of 30) but only 14% of F1 tumors (2 of 14) showed mutations in the CDR3-regions of amplified IgH-rearrangements (Table S3 and Table S4) (data for mutations in FR1-3 and CDR1–2 not shown).

### CD30^+^ Hodgkin/Reed Sternberg-Like Cells of B Cell Origin

**Figure 4:**
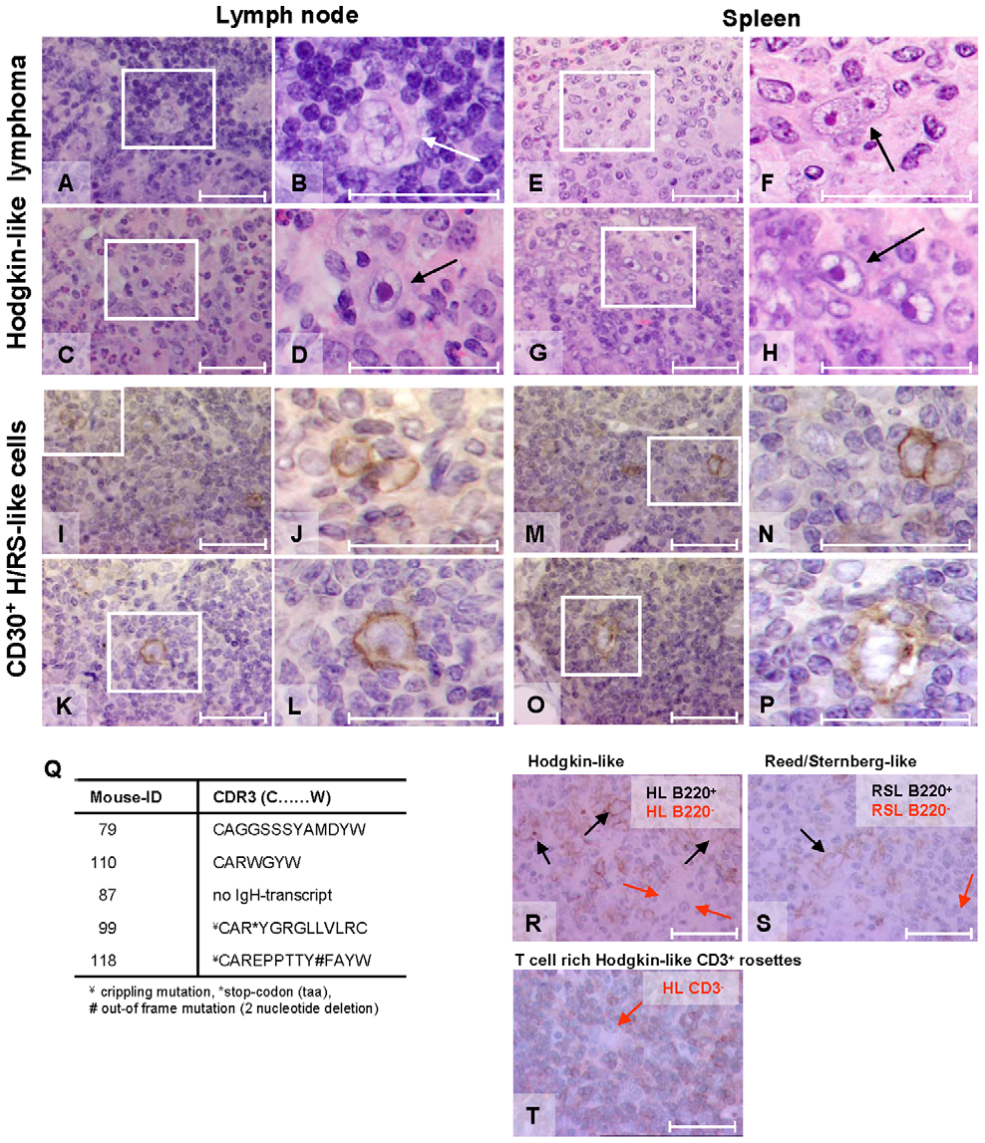
H/RS-like cells in composite lymphomas. (A-P+R-T) Histology and immunohistochemistry of Hodgkin-like lymphoma samples in composite compartments, surrounded by activated B and T cells as well as histiocytes. (A–H). H&E staining of (A–D) lymph node and (E–H) spleen sections containing Hodgkin/Reed-Sternberg (H/RS)-like cells (in white boxes). Magnification of characteristic (D+H) giant mononucleated Hodgkin-like and (B+F) multinucleated RS-like cells. (K+L & O+P) CD30+ Hodgkin-like and (I+J & M+N) RS-like cells in (I–L) lymph node and (M–P) spleen sections. (Q) CDR3-region of IgH-transcripts of microdissected H/RS-like cells from five different mice. (R–T) Immunohistochemistry of either B220 positive or negative (R) Hodgkin-like and (S) RS-like cells surrounded by (T) rosettes of CD3+ T cells. All bars equal 50 µm.

Unusual cells were found in the majority of composite lymphomas that were morphologically similar to human multinucleated Reed Sternberg cells or mononucleated Hodgkin cells (Figure 4A–H). These were designated as H/RS-like cells. A polymorphic infiltrate of B and T cells as well as histiocytes and plasma cells was detected surrounding the H/RS-like cells. Human H/RS cells are known to express CD30 molecules. Concordantly, murine H/RS-like cells in lymph node (Figure 4I–L) and spleen (Figure 4M–P) were also CD30 positive. We therefore aimed to determine whether these murine H/RS-like cells were of B cell origin and if characteristic mutations could be found in their IgH sequences. Such findings would further support their similarity to human HL. The B cell origin of murine H/RS-like cells from five mice was assessed using RT-PCR analysis of laser-assisted microdissected pools of 8–10 cells for each splenic tumor. Pooling of the cells was required to obtain adequate RNA from cells isolated from cryopreserved H&E sections that were identified without staining of specific markers but rather through morphologic distinction of H/RS-like cells. Sequence analysis was performed as described for B cell tumors above. Contaminating material from neighbouring normal B cells, if present, was below the threshold of detection in electropherograms.

Presence of IgH rearrangements showed that four tumors were indeed derived from B cells. Furthermore, amplicon sequence analysis and comparison with germline sequences (IMGT-Database) demonstrated the presence of mutations in the FR1–FR3 and CDR1–CDR3 regions of the specific amplicons, reflecting the occurrence of somatic hypermutation that occurs in germinal center (GC) B cells (Figure 4Q and data not shown). Interestingly, in addition to mutations in FR1–FR3 and CDR1–2 regions, two sequences displayed deleterious “crippling” mutations in their CDR3 regions, as has been reported for some human H/RS-cells [23], [24]. Based on IgH sequence comparisons of nucleotides inserted within the VD and DJ junctions, a clonal relationship was excluded between H/RS-like cells and tumor cells of DLBCL-HA or SMZL in the same mouse (Table S5 and Table S6).

Individual H/RS-like cells were positive or negative for B220 and were often surrounded by CD3^+^ reactive T cell rosettes (Figure 4R–T). Surface phenotypes of H/RS-like cells in composite tumors were unclear due to low frequencies (2–5%) of accompanying non-H/RS-like tumor cells (Table S7; File S1). Loss of expression of B cell markers, such as B220 on H/RS-like cells, was previously described for human H/RS cells [25]. In total, the characterization of these unusual cells strongly suggests that similar pathogenetic mechanisms may impact on the phenotype of H/RS cells in humans and mice.

### Impact of Transgene Integration

**Figure 5:**
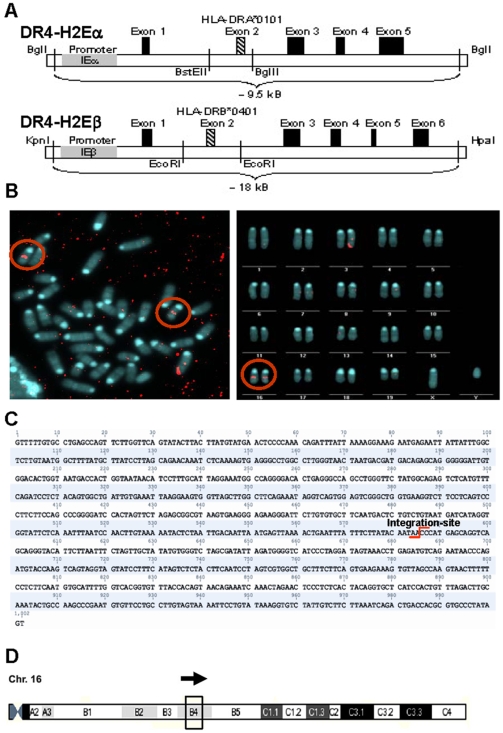
DR4-H2E constructs (DR4-a & DR4-b) and their genomic location after integration. (A) Schematic organization of the DR4-H2E constructs according to Ito et al. [12]. The endogenous exons 2 of DR4-H2Ea and DR4-H2Eb of the H2d-haplotype were replaced by the human exons 2 of HLA-DRA*0101 and HLA-DRB*0401, respectively in the described restriction enzyme sites of an SK+ vector. (B+C) Mapping of the DR4 transgene integration site. (B) left: Chromosomes from splenic metaphase; red circles mark the hybridization signals of a DR4-H2Ea-specific probe labeled with avidin-Cy3.5. Exemplary illustration of DR4-H2Ea in the genomic region between the cytogenetic banding B3 and C2, right: Diploid set of chromosomes arranged in a karyogramm. Both copies of chromosomes 16 show a hybridization signal (red circle) at the same genomic location. (C+D) Exact localization of transgene integration in Chromosome 16. (C) Fusion sequence of transgene integration. The 5′ end starts with the transgenic sequence of a DR4-α-construct marked in the middle is the integration site followed by the endogenous sequence of the DR4 mouse strain. Sequence was analyzed by using the NCBI-BLAST-software (http://www.ncbi.nlm.nih.gov/blast/) and determined at position +41138813 (Ensemble, www.ensemble.org). (D) Schematic diagram of Chromosome 16. The integration was localized to cytogenetic band B4 by genome walking analysis.

To determine whether transgene chromosomal integration disturbed a known murine locus with oncogenic or suppressor gene function, we sought to localize the exact insertion site. The chimeric class II genes were created by replacing exon 2 of the mouse gene with exon 2 of the corresponding human DR4 gene. The two chimeric constructs (Figure 5A), also comprising the locus control regions (LCR) of the mouse class II locus, were co-microinjected into C57BL/6 blastocysts. Therefore, the chimeric class II gene was expressed under the control of the natural murine class II promoter [12]. To determine the chromosomal integration site, we first performed fluorescence in-situ hybridization (FISH) using transgene-specific (DR4-exon2α and DR4-exon2β) probes on splenocytes in metaphase. As indicated in Figure 5B, the avidin-Cy3.5-labeled probe (DR4-exon2α) showed a strong signal on both copies of chromosome 16 (comparable data for DR4-exon2β not shown). The position of transgene integration was estimated on chromosome 16 to be near cytogenetic bands B3 to C2. The FISH analysis also revealed that integration of both the DR4-chimeric alpha and beta transgenes occurred together at a single location in the mouse genome. To further pinpoint the exact localization of this integration site, a genome walking analysis was performed using transgene specific primers combined with adaptor primers to “walk” from within the chimeric transgene to the murine chromosomal boundary region, yielding a fusion-sequence. This sequence contained DR4 transgene sequence at the 5′-end and a murine chromosome 16 sequence at the 3′-end (Figure 5C). The exact integration site was located by BLAST search of chromosome 16, at position +41138813 (Ensembl, www.ensembl.org), corresponding to cytogenetic band B4 (Figure 5D). To date no known genes have been annotated in the vicinity of this chromosomal region based on alignment with Ensembl-Database.

## Discussion

The development of diverse hematological malignancies in this DR4 transgenic mouse line represents a unique phenotype that has not been seen to date in other mouse tumor models. These animals spontaneously develop a variety of lymphoid malignancies in both the T and B cell lineages. Furthermore, the neoplasms represent different developmental stages of T cells (DP and SP T cells) and B cells (pre, pro, immature, mature, plasma cells). While the majority of malignancies were derived from the lymphoid lineage, the development of several cases of AML point towards the possibility that lymphoid, or even earlier precursor cells with lymphoid characteristics, occasionally transformed into myeloid tumors, as recently proposed by others [10]. Malignant transformation was strongly restricted to the hematopoietic lineage since only one case of carcinoma was observed in the more than 250 mice that underwent multi-organ histological examination. This probably reflects a high propensity for rapidly dividing hematopoietic cells to undergo malignant transformation.

The expression of only one transgenic chromosome 16 in heterozygous F1 mice was sufficient to allow development of similarly diverse neoplasms. Since only 70% of DR4 mice and 53% of F1 mice developed tumors, DR4-H2E transgene expression/integration alone was not sufficient for tumorigenesis. The observation that malignancy first appeared around eight months of age is consistent with a time-dependent, multi-step process of transformation in these mice. The primary hit may impact an early cell, perhaps a stem or early progenitor/precursor cell, since some myeloid malignancies were observed but the final events in tumorigenesis were only manifest at later stages of cellular development. A single transformation event, occurring in an early stem/precursor cell, seemed unlikely since this should lead to polyclonal tumors, whereas the vast majority of lymphomas were monoclonal with respect to TCR or IgH rearrangements. Nevertheless, the strong propensity for B and T cell lymphomas may be related to events occurring during Ig or TCR receptor rearrangement that contributed to a postulated accumulation of genetic hits needed for ultimate transformation. When tumors were first apparent they were very aggressive and caused fulminant and wide-spread disease, arguing against an early development of tumors that remained quiescent and unseen during the first months of life.

Among the diverse malignancies, the appearance of H-like and R/S-like cells in the compartment of T cell/histiocytic-rich DLBCL is of particular interest. Various investigators have attempted to establish murine models of HL by transplanting human HL cell lines or freshly isolated HL biopsies into immunodeficient SCID mice [26], [27], [28]. These models are limited by their xenogeneic nature, whereas the DR4 mice described here provide cases of spontaneous disease with morphological and phenotypic similarities to human T cell/histiocyte rich DLBCL and HL with characteristic H/RS-like cells. Single cell analysis of IgH-rearrangements in the H/RS-like cells revealed a B cell origin in four of five examined cases. Furthermore, “crippled” mutations in the hypervariable CDR3-regions suggested a pre-apoptotic germinal center B cell origin in at least two cases, as shown for some human H/RS cells [23], [24]. In most human composite lymphomas, a shared precursor for the HL and accompanying B cell tumor was demonstrated by presence of common Ig sequences and shared mutations [14], [15]. In contrast, sequence comparisons of the microdissected HL-like cells and the accompanying DLBCL-HA or SMZL in DR4 mice showed clonally unrelated V_H_, D_H_ and J_H_-families. Likewise, the group of Caleo described a composite human HL and mantle cell lymphoma as two clonally unrelated tumors [29]. Furthermore, comparison of IgH sequences with germline configurations revealed accumulation of mutations in framework (FR1–FR3) and complementarity-regions (CDR1–CDR3) of murine H/RS-like cells that were not detected in accompanying DLBCL-HA or SMZL (data not shown), revealing their origin from two distinct B cells. Nevertheless, the appearance of additional mutations outside the CDR3-region supports the conclusion that murine H/RS-like cells were derived from GC B cells and the crippled mutations seen in two cases were the result of ongoing somatic mutations rather than being due to aberrant VDJ recombination.

The cellular and molecular analyses of the DR4 mice provide substantial insight into the basis of tumor development. The genomic DR4-H2E transgenes used for creation of DR4 mice contained the murine class II promoter and locus control regions (LCR), enabling natural expression of the chimeric proteins in the appropriate cells and at the appropriate stages of development, as expected for a normal H2 class II molecule [12]. Thereby, the DR4-H2E transgene was not aberrantly expressed in time or place in a manner that could contribute to tumor development.

It cannot be ascertained whether the chimeric class II protein plays a direct role in lymphoma development since only one founder line was retained by the original investigators and is available for study today. To the best of our knowledge this type of lymphoma development is unique to these DR4 mice since a literature search did not reveal a similar phenotype in other MHC class II tg mice [30], [31]. However, long periods of observation may be needed to detect tumors. If the DR4-H2E chimeric protein is directly involved, its expression alone is not sufficient for tumor development because only 70% of homozygous DR4 mice developed tumors, as ascertained by monitoring of all DR4 mice throughout their entire natural lifespan. It remains to be determined whether the chimeric class II protein impinges on particular cellular interactions or functions that could contribute to malignant transformation. It should be noted that chimeric protein expression in tumor cells was not essential, since the T cell tumors were all class II negative (data not shown). H2 class II deficiency appeared not to be essential for tumorigenesis since heterogenous F1 mice were also affected, despite carrying one normal H2 haplotype. Since the frequency of tumors was lower in F1 compared to DR4 mice, further studies are required to determine if there is some subtle impact of H2 class II deficiency on tumor development.

At first, it seemed most plausible that transgene insertion impacted on one or several murine loci, causing oncogenic effects. The finding that integration of both constructs occurred at a single site ruled out multiple hits due to multiple transgene insertion sites. Furthermore, identification of the exact transgene integration site failed to reveal disruption of a known suppressor gene or activation of a potential oncogene since no annotated gene is located within 1 MB in both directions of the insertion site. However, it is possible that long-range effects on other loci, aberrant recombination events, or abnormal chromosomal pairing and segregation caused by transgene integration could contribute to tumorigenesis. It is also possible that impacts on non-coding sequences localized near the integration site could play a role in tumor development. The age-related appearance of tumors suggests a multi-step process. Future broad scale approaches, therefore, will be needed to further pinpoint the complex mechanisms contributing to lymphoma development.

In summary, this DR4 line is a unique lymphoma “generator” that yields diverse hematological malignancies. The majority of lymphomas, including the DP LTCL, SMZL and DLBCL-HA described in these mice, represent significant diseases in man, whereas most other naturally occurring hematological tumors in mice do not share major characteristics with human tumors [32]. The appearance of several cases of AML opens the door to better elucidate the origin of these leukemias in relation to lymphoid malignancies. Furthermore, the identification of neoplasms with CD30^+^ H/RS-like cells with a probable origin from GC B cells may contribute to development of a long-sought mouse model for human HL. Current xenogeneic models of HL are hampered by low engraftment efficiency and the need for immune suppression. In addition, these transplanted cells lack the characteristic morphological phenotype of human HL. Therefore, a number of features of the DR4 line make it a useful model to investigate common molecular mechanisms that may contribute to important neoplastic diseases in man. Based on the high frequency of spontaneous tumor development, this DR4 line can also be used to assess targeted therapies with potential for wide application in different hematopoietic malignancies.

## Material and Methods

### Mice

DR4 tg mice have a C57BL/6 background [12] and were a kind gift of Z. Nagy (Munich, Germany). Mice were bred and propagated under SPF conditions. Mouse studies were conducted according to German law by approved experimental protocols. C57BL/6 mice (Taconic) were crossed with DR4 to generate F1 mice. At first sign of disease, animals were euthanized and dissected organs prepared for histopathology, immunohistochemistry (formalin-fixed/paraffin-embedded or cryopreserved) and flow cytometry (single cell suspensions).

### Classification

The classification of lymphoid and non-lymphoid tumors was based on the Bethesda Proposals [1], [2] and was supported by parallel flow cytometry studies. More information is provided in supplementary data.

### Histopathology/Immunohistochemistry (IHC)

Formalin-fixed lymph nodes, spleen and other organs were embedded in paraffin, cut in 2–3 µm sections and stained with H&E. Automated immunohistochemical staining (Ventana Medical Systems) was performed as published [21] using the following primary antibodies: CD45R/B220 (BD), CD3, CD79a, Tdt (Dako), CD30 (Chemicon), CD49b (eBioscience), MPO (NeoMarkers). The L243 (HLA-DR) antibody was kindly provided by J.P. Johnson (Ludwig-Maximilians-Universität, Munich, Germany). Biotinylated goat anti-mouse (Dako) or goat anti-rabbit (Vector) IgG antibodies were used as secondary reagents, linked to a streptavidin-HRP-complex (Jackson Immunoresearch Laboratories) and visualized with diaminobenzidine (Sigma-Aldrich).

### Laser-Assisted Microdissection of Single Cells

Cryosections (12 µm) were transferred onto PEN-PALM membrane slides (P.A.L.M.) and stained with H&E. Microdissection was performed in areas containing one H/RS cell. Up to 8–10 H/RS-like cells per sample were transferred into PALM Adhesive Caps (P.A.L.M.) using the P.A.L.M MicroBeam instrument. The mRNA was isolated with the BioNobile Quick Pick mRNA-Isolation Kit (BioNobile) according to the manufacturer's protocol and further processed with the message Booster cDNA Synthesis Kit for qPCR (Epicentre).

### RNA

RNA isolation of single cell suspensions or 20 µm thick tissue-cryosections was performed using the RNeasy-Kit (QIAGEN) following the manufacturer's protocol. Total RNA (1 µg) was reverse transcribed utilizing the AMV-First Strand cDNA-Synthesis Kit (Roche).

### Clonality Analysis of B Cells

Amplification of IgH rearrangements was performed by RT-PCR on a LightCycler (LC) instrument (Roche) using the LC FastStart DNA Master^Plus^ SYBR Green I–Kit. Degenerated IgH forward 5′-AGGTC/GA/CAA/GCTGCAGC/GAGTCATGG-3′ and IgH reverse 5′TGAGGAGACGGTGACCGTGGTCCCTTGGCCCC-3′ were kindly provided by R. Mocikat (Helmholtz Zentrum München, Germany). The forward primer annealed to the V_H_-region, the reverse primer to the J_H_-region and yielded products of ∼330 bp that included the VDJ-junction. LC-PCR conditions used initial denaturation at 95°C for 10 min followed by 35 cycles at 95°C for 0 sec, annealing at 65°C for 25 sec and elongation at 72°C for 25 sec. Specific fragments were extracted with NucleoSpin Extract II-Kit (Macherey&Nagel) and sequenced (Sequiserve) afterwards. Obtained sequences were compared with germline configurations using IMGT/V-Quest software on the IMGT website (http://imgt.cines.fr/IMGT_vquest/share/textes/) [22], [33].

### Clonality Analysis of T Cells

TCR-Vβ-repertoire analysis was performed by RT-PCR on a Speed-Cycler instrument (Analytik-Jena) using a 5′ forward primer panel (21 Vβ-family primers) described previously [17]. The reverse TCR-Jβ primer containing a GC-clamp 5′-CGCCCGCCGCGCCCCGCGCCCGTCCCGCCGCCCCCGCCGGCTTGGGTGGAGTCACATTTCTC-3′ was kindly provided by R. Mocikat. In each PCR, 22 single reaction mixes were set up in a special 36-well-plate (Analytik-Jena). The volume of each single mix was 20 µL and contained all components except the primer (each 2.5 pmol) with 0.9 µL cDNA, 2 µL 10x buffer, 0.2 mM dNTPs, 0.5 mM MgCl_2_, 1 unit Taq polymerase and H_2_O. PCR conditions used initial denaturation at 94°C 2 min, followed by 40 cycles of denaturation at 94°C for 15 sec, annealing at 60°C for 25 sec, elongation at 72°C for 30 sec and a final elongation of 72°C for 5 min. Editable sequences were compared with germline configurations using IMGT/V-Quest software.

### Flow Cytometry Analysis of TCR-Vβ-Repertoires

TCR-Vβ surface expression was analyzed using a mouse Vβ TCR Screening Panel Antibody Kit including 15 monoclonal FITC-labeled antibodies (BD) as well as PE-labeled antibodies for Vβ8.1/8.2 and Vβ7, CD3-PacBlue, CD4-AlexaFl700, CD8-APC-Cy7, B220-PerCp-Cy5.5 (eBioscience). Staining was performed in the presence of Fc-receptor blocking antibody (clone 2.4G8 kind gift of E. Kremmer, Helmholtz Zentrum München). All cells were processed on a LSRII Flow Cytometer (BD) and analyzed with FlowJo8.7.1 software. Dead cells were excluded using propidium iodide labeling and duplets by gating on single cells.

### Confocal Microscopy

Cells were prepared as described for flow cytometry. Images were acquired on a Personal Deltavision Microscope (Applied Precision LLC). Image deconvolution was performed with a constrained iterative algorithm as described [34] and analyzed with SoftWoRx Suite software.

### FISH Analysis

FISH analysis was performed on chromosome metaphase spreads as described elsewhere [35], [36]. Probes for FISH analysis were specific for the human exon-2 sequence and were amplified with hEx2α forward 5′-GGGAAGCAGGGGGACTATGAC-3′ and reverse primers 5′-CATTGGTGATCGGAGTATAGTTG-3′ labeled with a Biochem-link kit from Roche followed by hybridization to chromosomal metaphase spreads of DR4 splenocytes. The transgenic chromosome(s) positive for DR4-H2Eα were tentatively identified by size and DAPI banding. Images were recorded with a motorized Zeiss Axioplan Imaging II microscope (Zeiss, Göttingen, Germany) and the Isis/V3.4.0-Software (Metasystems, Altlussheim, Germany).

### Genome Walking

Genome walking analysis was performed with the GenomeWalker Universal kit (BD Biosciences Clontech) according to the user's manual. Gene-specific primer (GSP) and adaptor primer (AP) for the DR4-H2Eα-construct were used as follows αGSP1 5′-GGAGACCTCATCTTCTTCAGTTTCCAG-3′, and αAP1 5′-GTAATACGACTCACTCACTATAGGGCACTATAGGGCACGCGTGGT-3′ for primary PCR and αGSP2 5′-GTTTTTGTGCCTGAGCCAGTTCTTGGT-3′, and αAP2 5′-ACTATAGGGCACGCGTGGT-3′ for secondary PCR.

### Accession Numbers

Sequence data of lymphoid receptors with the following accession numbers FM179543–FM179580, FM179582–FM179603 and FM179714–FM179741 can be accessed from EMBL Nucleotide Sequence Database.

## Supporting Information

Figure S1 Histological and immunohistological staining of composite and non-lymphoid tumors. (A–H) Composite tumors consisted of different types of lymphoid tumors (A–E) or a B cell tumor was associated with an AML (F–H). (A–D) The LTCL in thymus and lymph nodes (left side) is accompanied by a DLBCL in the spleen (right side). (C) The LTCL is CD3^+^ and (D) the DLBCL is positive for CD79a. (E) Both composite tumors in the spleen are of B-cellular origin: The DLBCL-HA on the left side can be distinguished from the splenic marginal zone lymphoma (SMZL) on the right side. (F–H) A composite tumor in the spleen, which consists of (G) a B220-positive diffuse large B cell lymphoma (DLBCL), and (H) an MPO-positive acute myeloid leukemia (AML). (I–L) The well-differentiated acute myeloid leukemia (AML) with maturation consists of myeloblasts (less than 90%) and mature granulocytes with typical doughnut-like shape. (I) H&E stained section, (J) Immunohistochemically, it is characterized by myeloperoxidase (MPO) positivity of the mature granulocytes, (K) B220- and (L) CD3- cells. (M) Histiocytic sarcoma in lymph node section. All bars equal 50 µm.
(1.41 MB TIF)

Table S1 T cell neoplasms in DR4 mice. Phenotypes of T cell tumors and their monoclonal TCRVβeta chain sequences (CDR3-region).
(0.37 MB TIF)

Table S2 T cell neoplasms in F1 mice. Phenotypes of T cell tumors and their monoclonal TCRVβeta chain sequences (CDR3-region).
(0.27 MB TIF)

Table S3 B cell neoplasms in DR4 mice. Phenotypes of B cell tumors and their rearranged IgH-V family sequences (CDR3-region).
(0.38 MB TIF)

Table S4 B cell neoplasms in F1 mice (no FACS data). Rearranged IgH-V family sequences (CDR3-region).
(0.14 MB TIF)

Table S5 HL-like tumors in DR4 mice (microdissected pooled single cells). Rearranged IgH-V family sequences (CDR3-region).
(0.11 MB TIF)

Table S6 Composite tumors in DR4 mice (whole spleen samples). Rearranged IgH–V family sequences (CDR3-region).
(0.13 MB TIF)

Table S7 Immunhistology and flow cytometry of B cell lymphomas.
(0.36 MB TIF)

File S1 Flow cytometric classification of tumors Cell staining was performed in the presence of Fc-receptor blocking antibody (clone 2.4G8, kind gift of E. Kremmer, Helmholtz Zentrum München) using the following antibodies: FITC-labeled anti-CD62L, -TCRba, -CD4, -IgD, -2.4G2, PE-labeled anti-TCRdg, -CD117 (ckit), -CD11c, -CD70, -CD122, -CD86, APC-labeled anti-CD44, -IgM, -CD49b, L243 (HLA-DR), APC-Cy7-labeled anti-CD19, anti-GR1, -CD8, PE-Cy7-labeled anti-CD25, anti-CD11b CD4-Ax700, B220-PerCpCy5.5, CD3-Pacific Blue. Flow cytometry analysis was performed on a LSRII Flow Cytometer (BD) and analyzed with FlowJo8.7.1-software. Dead cells were excluded using propidium iodide labeling and duplets by gating on single cells (FSC-H to FSC-A channel).
(0.03 MB DOC)
