# Supplementary figures and images for: Diverse Hematological Malignancies Including Hodgkin-Like Lymphomas Develop in Chimeric MHC Class II Transgenic Mice
Source: PLoS One. 2009 Dec 31;4(12):e8539. doi: 10.1371/journal.pone.0008539 (PMC2796171; doi:10.1371/journal.pone.0008539)

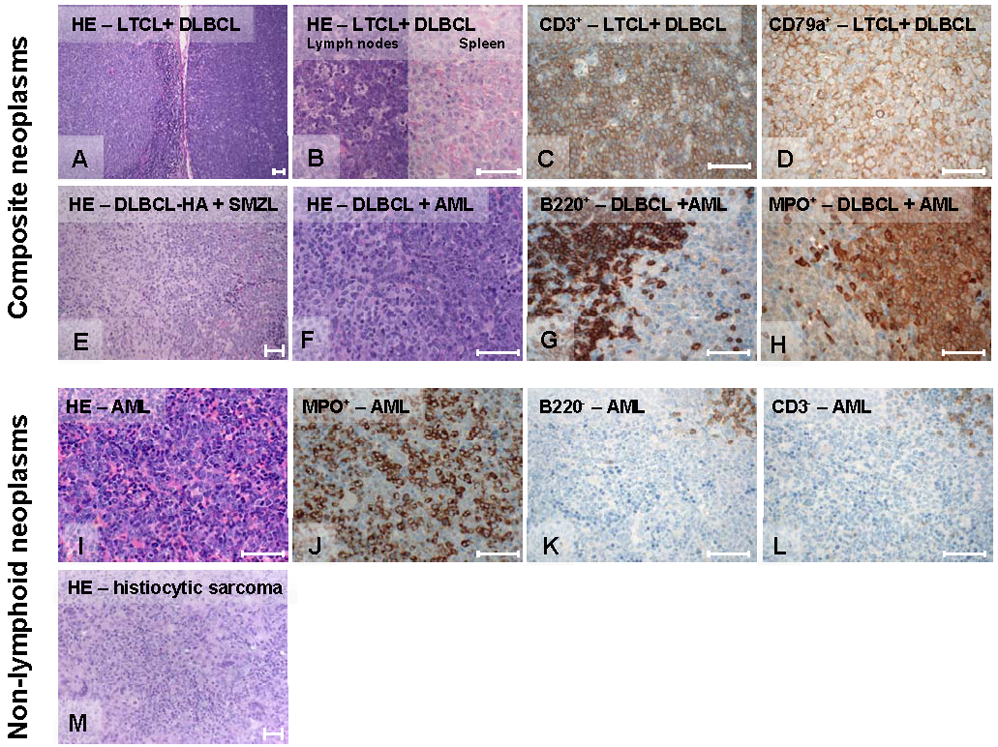

Supplement: Figure S1 — Histological and immunohistological staining of composite and non-lymphoid tumors. (A–H) Composite tumors consisted of different types of lymphoid tumors (A–E) or a B cell tumor was associated with an AML (F–H). (A–D) The LTCL in thymus and lymph nodes (left side) is accompanied by a DLBCL in the spleen (right side). (C) The LTCL is CD3+ and (D) the DLBCL is positive for CD79a. (E) Both composite tumors in the spleen are of B-cellular origin: The DLBCL-HA on the left side can be distinguished from the splenic marginal zone lymphoma (SMZL) on the right side. (F–H) A composite tumor in the spleen, which consists of (G) a B220-positive diffuse large B cell lymphoma (DLBCL), and (H) an MPO-positive acute myeloid leukemia (AML). (I–L) The well-differentiated acute myeloid leukemia (AML) with maturation consists of myeloblasts (less than 90%) and mature granulocytes with typical doughnut-like shape. (I) H&E stained section, (J) Immunohistochemically, it is characterized by myeloperoxidase (MPO) positivity of the mature granulocytes, (K) B220- and (L) CD3- cells. (M) Histiocytic sarcoma in lymph node section. All bars equal 50 µm. (1.41 MB TIF) [file pone.0008539.s001.tif]
