# Supplementary material for: Diverse Hematological Malignancies Including Hodgkin-Like Lymphomas Develop in Chimeric MHC Class II Transgenic Mice
Source: PLoS One. 2009 Dec 31;4(12):e8539. doi: 10.1371/journal.pone.0008539 (PMC2796171; doi:10.1371/journal.pone.0008539)
Supplement: File S1 — Flow cytometric classification of tumors Cell staining was performed in the presence of Fc-receptor blocking antibody (clone 2.4G8, kind gift of E. Kremmer, Helmholtz Zentrum München) using the following antibodies: FITC-labeled anti-CD62L, -TCRba, -CD4, -IgD, -2.4G2, PE-labeled anti-TCRdg, -CD117 (ckit), -CD11c, -CD70, -CD122, -CD86, APC-labeled anti-CD44, -IgM, -CD49b, L243 (HLA-DR), APC-Cy7-labeled anti-CD19, anti-GR1, -CD8, PE-Cy7-labeled anti-CD25, anti-CD11b CD4-Ax700, B220-PerCpCy5.5, CD3-Pacific Blue. Flow cytometry analysis was performed on a LSRII Flow Cytometer (BD) and analyzed with FlowJo8.7.1-software. Dead cells were excluded using propidium iodide labeling and duplets by gating on single cells (FSC-H to FSC-A channel). (0.03 MB DOC) [file pone.0008539.s009.doc]

**Supplemental Experimental Procedures**

**Flow cytometric classification of tumors**

Cell staining was performed in the presence of Fc-receptor blocking antibody (clone 2.4G8, kind gift of E. Kremmer, Helmholtz Zentrum München) using the following antibodies: FITC-labeled anti-CD62L, -TCRba, - CD4, -IgD, -2.4G2, PE-labeled anti-TCRdg, -CD117 (ckit), -CD11c, -CD70, -CD122, -CD86, APC-labeled anti-CD44, -IgM, -CD49b, L243 (HLA-DR), APC-Cy7-labeled anti-CD19, anti-GR1, -CD8, PE-Cy7-labeled anti-CD25, anti-CD11b CD4-Ax700, B220-PerCpCy5.5, CD3-Pacific Blue. Flow cytometry analysis was performed on a LSRII Flow Cytometer (BD) and analyzed with FlowJo8.7.1-software. Dead cells were excluded using propidium iodide labeling and duplets by gating on single cells (FSC-H to FSC-A channel).
